# The Collection and Application of Autologous Amniotic Fluid to Cesarean Delivery Closure

**DOI:** 10.1055/a-2445-7954

**Published:** 2024-11-18

**Authors:** Chad A. Grotegut, Kristin E. Weaver, Lena Fried, Sarah K. Dotters-Katz, Jennifer B. Gilner

**Affiliations:** 1 Division of Maternal-Fetal Medicine, Department of Obstetrics and GynecologyDuke University, Durham, North Carolina

**Keywords:** allograft, amniotic fluid, autologous, cesarean delivery, postsurgical adhesions, scar tissue, surgical site infection, wound complications, wound cosmesis, wound infection

## Abstract

**Background** Amniotic fluid and amnion membranes have been used in surgery specialties to improve wound healing and decrease surgical adhesion formation.

**Objective** The objective was to determine if amniotic fluid could be collected at cesarean delivery and then reapplied to the layers of the closure using the CeaLogic Specimen Collection and Ratio Applicator Kit.

**Study Design** Twenty pregnant individuals who met inclusion and exclusion criteria were enrolled. Amniotic fluid was collected at artificial rupture of the membranes using the collection kit. Autologous amniotic fluid was then transferred to the applicator kit, mixed with calcium chloride solution, and applied directly to each of the surgical repair layers during closure: closed hysterotomy incision, closed fascial incision, closed subcutaneous layer (if applicable), and closed skin. Subjects were then followed for six weeks. Photographs of the incision were taken immediately following surgery, one-week following surgery, and at the four-week postpartum visit. The Modified Hollander Cosmesis Score was used to assess wound appearance.

**Results** Twenty pregnant individuals who met inclusion and exclusion criteria were enrolled and all completed the study. The mean volume of amniotic fluid collected was 30 ± 19 mL. The median (IQR) Modified Hollander Cosmesis Score (Range 0-best, to 6-worst) at the one week and four-week postpartum visits was 0 (0,1) and 0 (0,2), respectively. There were no wound complications nor surgical site infections among the cohort. Further, there were no unscheduled visits for wound issues among any of the subjects.

**Conclusion** The CeaLogic Specimen Collection and Ratio Applicator Kits can be used to collect and reapply autologous amniotic fluid at the time of cesarean delivery. Future studies are needed to determine if the application of autologous amniotic fluid to cesarean delivery closure can improve cosmesis and wound healing, as well as decrease the risk for the development of intraabdominal adhesions.

## Introduction

Cesarean delivery is one of the most commonly performed surgical procedures in the United States, representing 32% of deliveries in 2021. 1 Each cesarean delivery places an individual at greater risk for repeat cesarean deliveries in future pregnancies as well as other future pelvic sugeries. 2 Cesarean deliveries are associated with the formation of scar tissue in the pelvis that can complicate future pelvic surgery, including repeat cesarean deliveries. 3 Adhesions of the pelvic organs from cesarean delivery make future pelvic surgery more difficult, increases surgical time and the risk for surgical complications including injury to internal organs, and also can result in chronic pain. 4 5 6 7

Processed amniotic fluid and amniotic membranes have been used in multiple surgical fields, including ophthalmologic, orthopaedic, and plastic surgery, as their use has been shown to potentially improve cosmesis and wound healing, and decrease the risk for scar formation and surgical adhesions. 8 9 10 11 12 13 Despite widespread use of processed amnion membranes and amniotic fluid preparations in other surgical specialties, the application of amniotic fluid to cesarean wound closure has not been reported.

Recibio, Inc (Houston, TX) has developed a device that allows for the efficient collection of amniotic fluid at the time of cesarean delivery. The collection kit pairs seamlessly with an applicator kit allowing for mixing with carrier or supplemental agents in a precise ratio, followed by immediate delivery as a spray of autologous amniotic fluid directed to the patient's tissues at the time of surgical closure. It is plausible that the application of autologous amniotic fluid to the various layers of the cesarean closure could improve wound healing and cosmesis as well as decrease the risk of intra-abdominal surgical adhesions. The objective of this study was to determine the feasibility of using the CeaLogic Specimen Fluid Collection and Ratio Applicator Kits for the collection of amniotic fluid at cesarean delivery and the subsequent application of autologous amniotic fluid to the various layers of the cesarean closure.

## Materials and Methods

We conducted a prospective cohort study to determine the feasibility of using the CeaLogic Specimen Collection and Ratio Applicator Kits to sterile collect and then apply autologous amniotic fluid to the layers of a cesarean delivery closure. The study was approved by the Duke University Health System (DUHS) Institutional Review Board (DUHS IRB# Pro00102749). The objective of this study was to determine if amniotic fluid could be collected at the time of cesarean delivery and then reapplied to the various layers of the cesarean closure as a mixture with calcium chloride. Completion of this feasibility study could then inform future studies designed to determine if autologous amniotic fluid could improve wound cosmesis and decrease adhesion formation, as well as decrease the risk for injury to internal organs during subsequent cesarean deliveries.

English-speaking pregnant individuals, age 18 years or greater, carrying a singleton fetus, who planned cesarean delivery at or greater than 37 weeks of gestation were eligible for participation. The study was designed to test feasibility of the device to collect and reapply amniotic fluid to the cesarean closure among a pregnant population who were at average risk for surgical site infection. Exclusion criteria at enrollment included body mass index greater than or equal to 40 kg/m ^2^ , diabetes requiring treatment (type I diabetes, type II diabetes, or gestational diabetes requiring medical management), abnormal placentation (placenta previa or placenta accreta spectrum), prior bowel or urological surgery except unruptured appendectomy or cholecystectomy, previous history of postpartum hemorrhage, tobacco or drug use, known or suspected impairment of immunologic function, infection with HIV, hepatitis B or C, history of keloid formation, or any condition, which in the opinion of the investigator, may pose a health risk to the subject. Following enrollment, study staff then assessed subjects again for potential exclusion criteria just prior to and at the time of surgery. The exclusion criteria at the time of surgery included labor at time of presentation to the labor and delivery unit (defined as regular, painful uterine contractions occurring every 5 minutes or more frequent with evidence of cervical change), chorioamnionitis, systemic infection, evidence of cutaneous candidiasis at the planned surgical incision, need for urgent cesarean delivery, rupture of the membranes prior to the start of surgery, intraoperative hemorrhage, or other medical condition during the delivery deemed by the investigator to pose a high probability of need for surgical reexploration or wound complication, need for vertical skin incision, intraoperative use of a hemostatic agent, plan for use of staples at skin closure, or preeclampsia with severe features. As this was a feasibility study, the study planned to enroll 20 participants and be stopped early if there were four (20%) wound complications (separations, seromas, hematomas) or surgical site infections that occurred.

The study was conducted between May 23, 2020 and August 3, 2022 at the Duke Birthing Center of the Duke University Hospital, Durham, NC. Subjects planning a cesarean delivery who met inclusion and exclusion criteria were approached by a study team participant and written consent was obtained. At the time of the cesarean delivery, a study team member was again present to determine if any additional exclusion criteria were present. All cesarean deliveries were performed by one of four physician investigators.

**Fig. 1:**
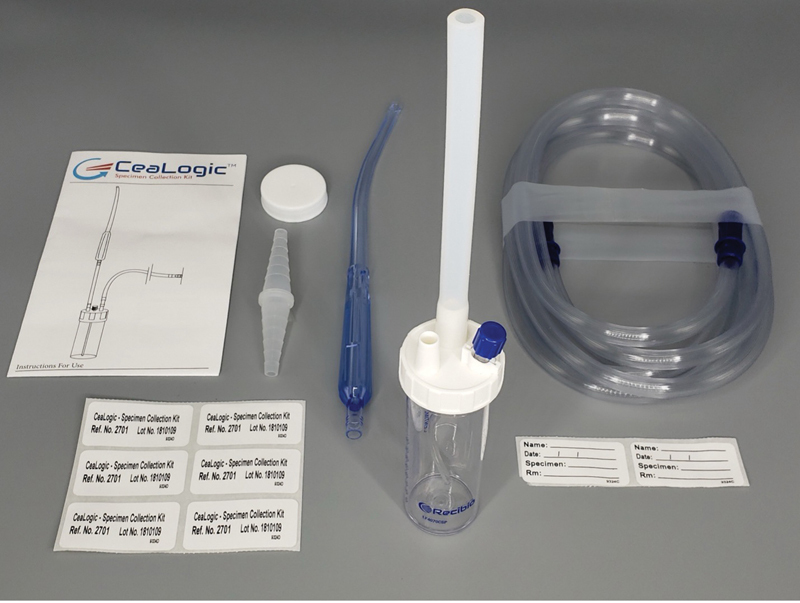
The Recibio CeaLogic Specimen Collection Kit. It includes a Yankauer suction tip, tubing, and an 80 mL collection trap with vacuum attachment.

**Fig. 2:**
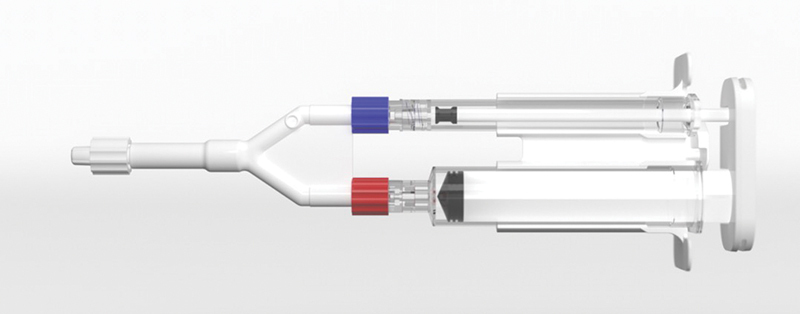
The Recibio CeaLogic Ratio Applicator Kit. It includes a 10-mL syringe, a 1-mL syringe that allows for the coadministration of a second fluid, and sprayer device that allows for the precise ratio mixture of the two applied fluids.

Following hysterotomy, the CeaLogic Specimen Fluid Collection device ( Fig. 1 ) was used to collect amniotic fluid at the hysterotomy site until either the collection trap was full (80-mL trap volume) or no further amniotic fluid was available for collection. Following delivery of the baby, placenta, and fetal membranes, the collected amniotic fluid was transferred from the collection trap to a 10-mL syringe, and the syringe was attached to the CeaLogic Ratio Applicator Kit ( Fig. 2 ). The CeaLogic Ratio Applicator Kit also includes a separate 1-mL syringe that was filled with 10% calcium chloride. 14 Approximate 2.5 mL of amniotic fluid with 0.25 mL of 10% calcium chloride (final calcium chloride concentration 1%) was then applied to each of the following four layers immediately following surgical closure using the applicator kit and sprayer: the closed hysterotomy incision, the closed abdominal fascial incision, the closed subcutaneous layer (if applicable), and the closed skin layer. The skin incision was then covered with a sterile surgical dressing. The sterile surgical dressing was then removed 24 hours following completion of the surgery, as per the standard practice at The Duke Birthing Center of Duke University Hospital.

Photographs of the incision were taken immediately at the completion of the procedure and then at the 1-week (postoperative day 6–8) and 4-week postpartum visits. At the 1- and 4-week postoperative visits, the following assessments were made by the study staff: (1) presence of any redness, swelling, or induration, (2) pain with rest and with gentle pressure, (3) medication use to include nonsteroidal anti-inflammatory agents and narcotics, (4) instructions on the use of a memory aid and pain scale. In addition, the study staff assessed the cosmesis of the wound using the Modified Hollander Cosmesis Score. 15 16 17 The Modified Hollander Cosmesis Score includes six components that are each graded as a 0 (being absent) or 1 (being present). The six components include: step-off borders (edges not on same plane), contour irregularities (wrinkled skin near wound), margin separation (gap between sides), edge inversion (wound not properly everted), excess distortion (swelling/edema/infection), and overall appearance (satisfactory vs. unsatisfactory). The six components are then added together to provide a total cosmetic score (0–6) with 0 being the best cosmetic score and 6 the worst. 15 16 17 At each study visit, the study team also inquired as to whether the participant had any wound issues or complaints and if they sought any medical care for their wound. Finally, 6 weeks following the procedure, the study team conducted a phone interview to determine if the subject had any wound complications since their last in-person visit that occurred at 4-week postpartum.

There were no costs to the subjects for participation, and subjects were compensated $25 following complication of the 1-week postoperative visit and $25 following completion of the 4-week postpartum visit. There was no comparison group as this was a feasibility study to test whether the device could be used to collect and then reapply amniotic fluid to the cesarean closures.

## Results

Twenty pregnant individuals who met inclusion and exclusion criteria were enrolled and all completed the study. No enrolled participants met exclusion criteria at the time of their cesarean delivery. Table 1 provides demographic information on the pregnant subjects included in the study. The mean age of the participants was 32.7 years (± 6.0) and mean body mass index at delivery was 28.8 kg/m ^2^ (± 3.4) ( Table 1 ). Fifteen (75%) of subjects were parous. The majority of the subjects were non-Hispanic White (65%), with non-Hispanic Black and Hispanic participants representing 15 and 10% of the population, respectively ( Table 1 ). The mean gestational age of delivery was 38 ^6/7^ weeks and 13 (65%) had had a prior cesarean delivery ( Table 2 ).

**Table 1:** Subject characteristics

Characteristic	CeaLogic—Duke cohort ( *n* = 20)
Age, y	32.7 ± 6.0
Race/ethnicity, *n* (%)	
White	13 (65)
Black	3 (15)
American Indian	0 (0)
Asian	1 (5)
Native Hawaiian/Pacific Islander	0 (0)
More than one race	1 (0)
Unknown	2 (10)
Ethnicity	
Hispanic/Latina	2 (10)
Non-Hispanic/Non-Latina	17 (85)
Unknown/not reported	1 (5)
Body mass index, prepregnancy, kg/m ^2^	24.6 ± 4.0
Body mass index, at delivery, kg/m ^2^	28.8 ± 3.4
Parous, *n* (%)	15 (75)

**Table 2:** Delivery characteristics

Characteristic	CeaLogic—Duke cohort ( *n* = 20)
Gestational age at delivery, wk	38w 6d
Prior cesarean delivery, *n* (%)	13 (65)
Pfannenstiel skin incision, *n* (%)	20 (100)
Investigator performing procedure	
Investigator #1, *n* (%)	5 (25)
Investigator #2, *n* (%)	10 (50)
Investigator #3, *n* (%)	4 (20)
Investigator #4, *n* (%)	1 (5)
Volume amniotic fluid collected, mL	30.0 ± 18.7
Amniotic fluid collection time < 1 min, *n* (%)	20 (100)
Amniotic fluid color	
Clear, *n* (%)	18 (90)
Blood tinged, *n* (%)	2 (10)
Spray volume to closed hysterotomy, mL	2.4 ± 0.6
Spray volume to closed fascia, mL	2.8 ± 1.1
Spray volumed to closed subcutaneous fat, mL	2.3 ± 0.4
Spray volume to closed skin, mL	3.0 ± 1.2
Depth of adipose layer, cm	1.8 ± 0.6
Adipose layer closed, *n* (%)	17 (85)
Quantitative blood loss, mL	497 ± 482
Duration of surgical procedure, min	61.6 ± 18.7
Length of closed skin incision, cm	14.4 ± 0.8

**Fig. 3:**
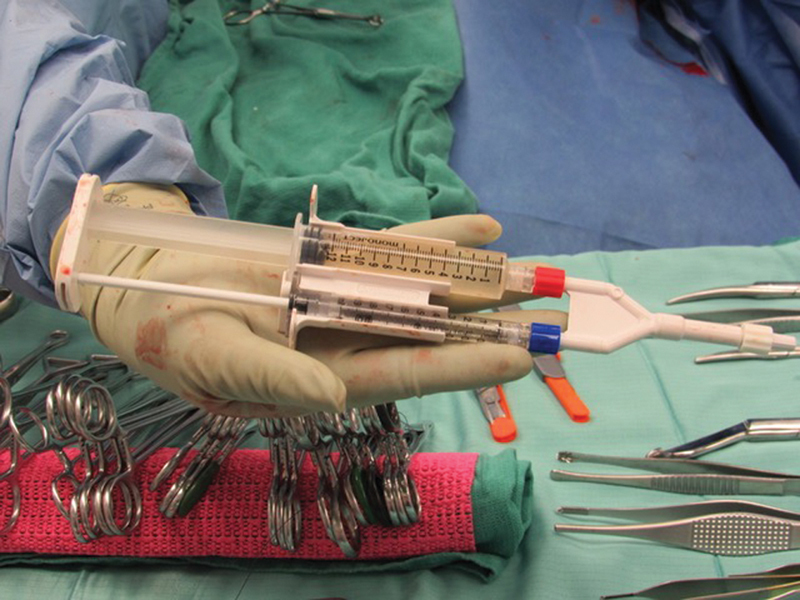
CeaLogic Ratio Applicator Kit immediately prior to application. Representative image of the CeaLogic Ratio Applicator Kit containing 10 mL of amniotic fluid and 1 mL 10% calcium chloride immediately prior to application to the cesarean layers.

Amniotic fluid was successfully collected and applied to the various layers of the cesarean closure in all study participants ( Fig. 3 ). The mean volume of amniotic fluid collected was 30 ± 19 mL and the collection time for all cases was less than 1 minute ( Table 2 ). There were no cases of meconium-stained or grossly bloody amniotic fluid. The mean depth of the adipose layer was 1.8 mm (± 0.6) and 17 (85%) of the subjects had the subcutaneous adipose layer closed. Table 2 provides data on the volume of amniotic fluid that was applied to each of the four layers of the closure, with a range of 2.3 to 3.0 mL to each of the four layers.

**Fig. 4:**
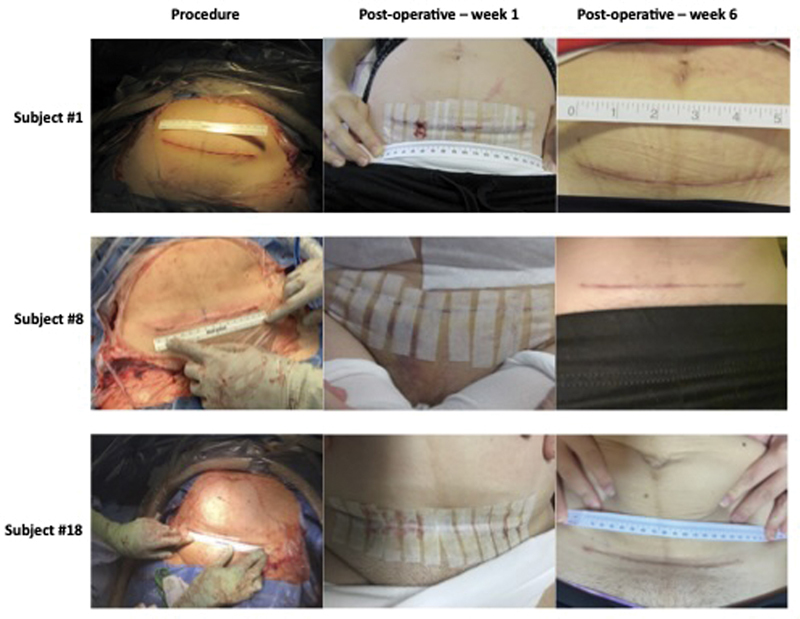
Representative images of postsurgical wound at time of procedure, 1-week postoperative, and 4-week postoperative. Representative images of the postoperative wounds from three subjects at time of procedure and at their 1- and 4-week visits.

At both the 1-week postoperative visit and the 4-week postpartum visit, the study team used the Modified Hollander Cosmesis Score to evaluate the appearance of the wound. 15 16 17 The median (interquartile range) Modified Hollander Cosmesis Score was 0 (0, 1) and 0 (0, 2) at the 1- and 4-week visits, respectively ( Table 3 ). There were no wound complications nor surgical site infections noted at the 1- and 4-week visits among the cohort. Fig. 4 provides representative images for cesarean wounds from three subjects immediately after the procedure and at their 1- and 4-week postpartum visits. None of the subjects required nonscheduled evaluation of their wound within the 6-week follow-up period ( Table 3 ).

**Table 3:** Wound and pain characteristics

Characteristic	CeaLogic—Duke cohort ( *n* = 20)
1-wk visit	
Hollander Cosmesis Score, median (range)	0 (0, 1)
Pain and Medication Questionnaire	
Worst pain level since the surgery, median (IQR)	70 (58, 83)
Current pain at rest	12 (2, 20)
Current pain with gentle pressure	28 (8, 36)
How satisfied with your pain control since the surgery	87 (61, 99)
Subjects with ED visit since discharge, *n* (%)	0 (0)
Subjects with wound complication since discharge, *n* (%)	0 (0)
Postpartum visit (4 wk)	
Hollander Cosmesis Score, median (range)	0 (0, 2)
Pain and Medication Questionnaire	
Worst pain level since the surgery	64 (44, 77)
Current pain at rest	0 (0, 7)
Current pain with gentle pressure	11 (0, 31)
How satisfied with your pain control since the surgery	98 (78, 100)
Subjects with ED visit since discharge, *n* (%)	0 (0)
Subjects with wound complication since discharge, *n* (%)	0 (0)
6-wk phone visit	
Subjects with ED visit since discharge, *n* (%)	0 (0)
Subjects with wound complication since discharge, *n* (%)	0 (0)

Abbreviation: IQR, interquartile range.

The physicians reported no difficulties with the use of the collection and applicator devices, except that in one case, vernix within the amniotic fluid slightly clogged the collection suction tubing, resulting in a somewhat slower rate of collection of amniotic fluid, but the complete collection was still able to be completed within 1 minute.

## Discussion

Cesarean delivery leads to the development of intrabdominal adhesions in many individuals, which potentially could complicate subsequent abdominal surgeries via increased risk for injury to intra-abdominal organs, increased surgical times, and can also result in chronic pain. 3 4 5 6 7 Amnion membranes and amniotic fluid preparations have been used in nonobstetric surgical specialties to promote wound healing and decrease scar formation, but the amniotic fluid and amnion membrane preparations are not used in an autologous fashion, therefore require donation from pregnant people, then processing and storage prior to use. 8 9 10 11 12 13 18 19 20 The collection and application of autologous amniotic fluid at cesarean delivery to the cesarean closure layers has not previously been reported. Results from this study can inform future studies that test the effectiveness of autologous amniotic fluid in improving long-term outcomes following cesarean delivery.

This feasibility study demonstrated that the CeaLogic Specimen Fluid Collection and Ratio Applicator Kits could be used to both collect and then reapply autologous amniotic fluid to all layers of the cesarean delivery closure. No wound complications occurred in this feasibility study and the use of the device did not significantly increase surgical times. In addition, there were excellent Modified Hollander Cosmesis Scores seen across the population at both the 1- and 4-week postoperative visits. A prior randomized trial of different subcutaneous closure methods that was also conducted at the Duke University reported similar Modified Hollander Cosmesis Scores following cesarean. 17

This is the first known study to report the collection and application of autologous amniotic fluid to the closure layers at time of cesarean delivery. In contrast, the use of processed stored amnion membranes and amniotic fluid has been reported extensively in nonobstetric surgical fields and some studies suggest that the use of processed amniotic membranes or amniotic fluid may improve surgical outcomes as well as improve wound healing in both acute and chronic wounds. 20 21

Although still considered experimental, the use of amniotic fluid and amnion membrane preparations have shown promise in multiple medical and surgical indications, and there has been significant growth in the industry that collects, processes, and supplies amnion membranes and amniotic fluid. 22 23 24 Companies currently provide cryopreserved or dehydrated amnion membranes for multiple indications, and there is expected significant growth in the market size as studies show favorable outcomes. 22 23 24 Ophthalmologic indications are currently the most prevalent use of amnion membrane preparations, with ocular surface reconstruction being the most common use in that field. 25 26 27

In addition to ophthalmologic uses, other uses of amnion membranes show promise and increasing utility. A recent meta-analysis in subjects with chronic diabetic foot ulcers, which included five randomized controlled trials, found that the use of processed human amnion membranes improved wound healing compared with controls. 18 Furthermore, a systematic review of wound healing in acute burn subjects reported that processed human amnion membranes may provide potential healing benefit. 19 In addition, amnion membrane and amniotic fluid preparations have been reported extensively in the sports medicine and orthopaedic literature including their use for cartilage restoration, nonoperative treatments for arthritis, and as an adjunct in tendon and ligament repair and replacement.

Despite potential benefits seen in various medical and surgical uses, the mechanisms by which amnion membrane and amniotic fluid preparations improve wound healing is not fully understood. Animal- and laboratory-based studies have shown that amnion membranes and amniotic fluid have antimicrobial properties, which may contribute to its ability to improve wound healing. 28 29 30 31 Additionally, amniotic fluid contains stem cells and anti-inflammatory cytokines, which may also improve wound healing and decrease fibrosis and scar formation. 32 33 34 35

The use of amnion membrane and amniotic fluid preparations in medical and surgical settings shows substantial promise. The autologous collection and application at the time of cesarean delivery is a unique opportunity that bypasses problems with tissue collection, preparation, and storage that complicate the application to nonobstetric indications. Future studies are needed in obstetrics to determine if autologous amniotic fluid application to the cesarean delivery layers improves surgical outcomes and decreases scar formation. Should autologous amniotic fluid indeed improve cesarean wound healing and decrease pelvic adhesions, the impact would be substantial.

This feasibility study was designed to determine the feasibility of utilizing a commercially available fluid collection and application kit and was not designed to demonstrate safety, nor the impact of autologous amniotic fluid on long-term postsurgical outcomes. It is reassuring that no wound complications occurred, but the application of autologous amniotic fluid to the wound closure layers would not have been expected to increase wound complications as amniotic fluid always spills into the surgical field at the time of cesarean delivery. Although beyond the scope of the current study, future studies can now be designed to determine if application of autologous amniotic fluid can improve wound cosmesis and long-term postsurgical outcomes.

In summary, the CeaLogic Specimen Fluid Collection and Ratio Applicator Kits can be used to collect and apply autologous amniotic fluid to cesarean layer closures. This study demonstrated feasibility of the device for this application and future studies can be designed to demonstrate the potential efficacy of autologous amniotic fluid in preventing long-term postsurgical complications associated with cesarean delivery.
