# Corrigendum: The Collection and Application of Autologous Amniotic Fluid to Cesarean Delivery Closure

**DOI:** 10.1055/s-0044-1801260

**Published:** 2024-12-17

**Authors:** Chad A. Grotegut, Kristin E. Weaver, Lena Fried, Sarah K. Dotters-Katz, Jennifer B. Gilner

**Affiliations:** 1 Division of Maternal-Fetal Medicine, Department of Obstetrics and Gynecology, Duke University, Durham, North Carolina

The authors have brought to the publisher’s attention about the error in one of the column labels in Figure 4 of the above article published on November 18, 2024, in Volume 14, Number 4 in the *American Journal of Perinatology Reports* (DOI: 10.1055/a-2445-7954 ).

The corrected Figure appear as below.



